# A Novel Solid State Non-Dispersive Infrared CO_2_ Gas Sensor Compatible with Wireless and Portable Deployment

**DOI:** 10.3390/s130607079

**Published:** 2013-05-29

**Authors:** Desmond Gibson, Calum MacGregor

**Affiliations:** Gas Sensing Solutions Ltd, 60 Grayshill Road, Westfield North Courtyard, Glasgow G68 9HQ, UK; E-Mail: calum@gassensing.co.uk

**Keywords:** CO_2_ sensor, energy harvesting, demand control ventilation, indoor air quality, portable gas sensors, mid infrared light emitting diodes, mid infrared photodiodes

## Abstract

This paper describes development of a novel mid-infrared light emitting diode (LED) and photodiode (PD) light source/detector combination and use within a non-dispersive infrared (NDIR) carbon dioxide gas sensor. The LED/PD based NDIR sensor provides fast stabilisation time (time required to turn on the sensor from cold, warm up, take and report a measurement, and power down again ≈1 second), longevity (>15 years), low power consumption and low cost. Described performance is compatible with “fit and forget” wireless deployed sensors in applications such as indoor air quality monitoring/control & energy conservation in buildings, transport systems, horticultural greenhouses and portable deployment for safety, industrial and medical applications. Fast stabilisation time, low intrinsic power consumption and cycled operation offer typical energy consumption per measurement of mJ's, providing extended operation using battery and/or energy harvesting strategies (measurement interval of ≈ 2 minutes provides >10 years operation from one AA battery). Specific performance data is provided in relation to measurement accuracy and noise, temperature performance, cross sensitivity, measurement range (two pathlength variants are described covering ambient through to 100% gas concentration), comparison with NDIR utilizing thermal source/pyroelectric light source/detector combination and compatibility with energy harvesting. Semiconductor based LED/PD processing together with injection moulded reflective optics and simple assembly provide a route to low cost high volume manufacturing.

## 1. Introduction

There is a growing need [1] for low power consumption, fast stabilizing (minimal time required to turn on the sensor from cold, warm up, take and report a measurement, and power down) wireless compatible carbon dioxide (CO_2_) gas sensors, driven by current and incoming legislation requirements [2,3] for CO_2_ gas monitoring/control within building and transport environments. Such autonomous CO_2_ sensors, powered from extended battery life/energy harvesting sources, provide a lower cost route to flexible deployment compared with hard wiring of CO_2_ sensors into building or transport systems infrastructures.

A primary need for CO_2_ sensors is for optimal control of indoor air quality (IAQ) [4,5] and use to reduce energy usage in heating, ventilating & air conditioning (HVAC) systems [6,7]. CO_2_ gas is safe in low concentrations (typically <1,000 ppm), however prolonged exposure at moderate levels (>5,000 ppm) can lead to a range of health related problems such as sick building syndrome [8,9] causing fatigue like symptoms. Use of CO_2_ concentration monitoring/control provides a means of providing IAQ which can be set to suit level of people occupancy through demand control ventilation (DCV) [6,7]—human exhaled air is rich in carbon dioxide gas, waste product of cellular respiration, and CO_2_ gas concentration monitoring provides an accurate means of determining people occupancy in a known volume.

In DCV, CO_2_ sensors, normally integrated with temperature and humidity sensing, allow the HVAC system to adjust the amount of outside air coming in based on the levels of CO_2_ in the building or transport system.

From an energy-efficiency standpoint, control should bring in as little outdoor air as possible. DCV with CO_2_ sensors provides a means of adjusting ventilation rate to suit the changing needs of the space and thus saving energy costs during times of lower occupancy levels. Reviews of the research literature on DCV [6,7], indicates a significant potential for energy savings, particularly in buildings or spaces with a high and variable occupancy. DCV benefits [6] in term of energy efficiency have demonstrated up to 25% of energy savings in buildings can be achieved with proper ventilation monitoring and control whilst achieving IAQ levels suited to occupant comfort and productivity.

As commercial buildings are responsible for at least 40% of the world's total energy consumption [10], with 96% of existing building stock currently with limited or no effective building energy management systems in place [10], use of DCV with CO_2_ sensing provides a route to achieving significant global energy savings.

Current deployment and powering of carbon dioxide sensors into building and transport environments for IAQ and/or DCV is normally through wired connection to the building/transport system electrical supply. However, use of autonomous wireless CO_2_ sensors is growing by typically four times that of wired sensors [1], on the basis of install flexibility and lower installation cost.

Moreover, use of wireless sensor networks (WSNs) is also increasing, enabling simultaneous, real time, high-speed sensing and data acquisition from multiple sensors located in various parts of the building for zoned control of CO_2_/humidity/temperature. Consequently, WSN implementation is also driving the need for low cost flexible deployment of CO_2_ sensors.

Deployment of autonomous CO_2_ sensors is also growing in horticultural applications, specifically within greenhouses. In particular deliberate introduction of CO_2_ into greenhouse environments is a well-known method to accelerate plant growth by as much as 25 to 40% [11] and high level exposure [typically 1%] is also used as a means of eliminating pests such as whiteflies and spider mites [12]. Temperature and humidity control are also required to provide optimal conditions for plant growth.

In addition to IAQ and DCV in buildings, transport and horticulture, other applications require monitoring of CO_2_ concentration level, such as industrial, automotive (IAQ/DCV as a route to anti-drowziness monitoring) [13] and medical/recreational sports (capnography for breath rate monitoring, respiratory condition monitoring/diagnostics and assessment of metabolic rate/energy usage) [14].

In industrial environments where process generated CO_2_ dominates, for example in breweries, soft drinks, packaging industry, freezer storage *etc.*, the maximum permitted CO_2_ concentration according to most standards can be as high as 5,000 ppm during an 8-hour working period. CO_2_ gas monitoring in this case is required to ensure air quality monitoring/control and compliance with industrial health and safety requirements.

These markets in addition to IAQ and DCV are creating high volume demand for low cost autonomous CO_2_ gas sensors. Moreover, usage within WSN platforms is also placing emphasis on cycled powering/intermittent use of sensors as a route to reducing power consumption, requiring fast stabilization times for sensors powered from cold, warm up, take and report a measurement, and power down. Primary aim is to achieve sensor power consumption/operating methodology at levels compatible with extended battery life operation and ultimately power scavenging/self-powering. Moreover, sensor portability required in many industrial safety and medical applications is also driving the need for autonomous low power consumption/extended battery operation.

Currently there are primarily two types of commonly available gas sensors for monitoring CO_2_ concentrations in air, *i.e.*, non-dispersive infrared (NDIR) [15] and solid electrolyte sensor [16] types. NDIR sensors have performance advantages in terms of long-term stability, accuracy, and power consumption for CO_2_ measurement. Hence, NDIR sensors are the most widely used for the real-time measurement of carbon dioxide. Solid electrolyte types have cost advantages, but the product performance is not good enough to allow their use in any but the least demanding applications.

The NDIR method is an optical method of detecting gases, relying on the fact that many gases absorb specific wavelengths of infra-red light [17,18]. It is possible to calculate the gas concentration, by passing light through a defined length and measuring how much light is absorbed at the specific wavelength absorbed by the gas. For CO_2_, the commonly used wavelength is 4.26 μm, which is not absorbed at all by other commonly found gases or by water vapour.

Current NDIR CO_2_ gas sensors utilise thermal sources (typically a tungsten filament lamp) and pyroelectric or thermopile light source/detector combination. Thermal sources have restrictions for use in wireless IAQ, DCV and portable applications due to high power consumption, incompatible with extended battery operation and/or energy harvesting. Moreover, cycled power/intermittent use operation increases power consumption due to extended stabilisation time (typically 1 to 10 minutes). Consequently thermal source-based NDIR CO_2_ sensors usually require hard wired installation or offer short battery life, which is costly and at odds with legislatively driven requirements [1,2].

Previous work published in 1997 [19], provides a comparison of gas sensor performance using mid-infrared light emitting diodes (LED) with thermal sources. Whilst the work described in [19] demonstrated the clear advantages using the LED light source, problems with early LED devices in relation to power output, temperature stability, lifetime and cost resulted in marginal performance improvements compared to thermal sources.

This paper describes an all solid state, low power consumption, reliable and low cost NDIR CO_2_ gas sensor, incorporating LED light sources and photodiode (PD) detectors [20,21], combined with state of the art signal processing matched to use of LED/PD's. LED/PD offer the advantage of low power consumption, high source emittance, fast modulation rates, room temperature operation, fast stabilisation time and brings with it the cost benefits of semiconductor manufacturing techniques [22].

## 2. Experimental

### 2.1. Sensor Configuration

The NDIR sensor described in this paper comprises a narrow bandgap III-V LED light source and photodiode detector. Mid-infrared radiation is launched into an optical structure which defines the lightpath and a chamber into which gas diffuses, causing a reduction in light transmission at the sensing wavelength. Electronics and intrinsic firmware control LED/PD drive current/voltage, pulsing and signal processing. Output parameter is CO_2_ gas concentration, stabilised over the sensor operating temperature range. Sensitivity is determined by the optical pathlength.

**Figure 1.**
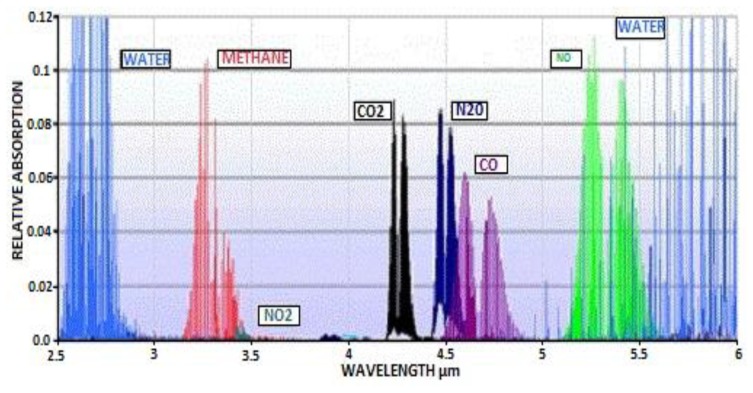
Absorption spectra for commercially relevant gases.

Typical mid-infrared absorption spectra [17,18] for a range of commercially relevant gases are shown in Figure 1 (note Figure 1 does not provide actual absorption cross-section data. However, indicated spectral position is representative of actual absorption bands).

It is possible to calculate the gas concentration by passing light through a defined length and measuring how much light is absorbed at the specific wavelength absorbed by the gas.

NDIR sensors are spectroscopic devices that can be applied to gas analysis. Typically, the main components of NDIR are infrared sources (lamps), sample chambers (or light tubes), wavelength filters, and infrared detectors. The gas is pumped (or diffused) into the sample chamber, and the concentration of target gas is measured electro-optically by its absorption of a specific wavelength in the infrared (IR) range. The IR light is directed through the sample chamber towards the detector.

An optical filter is normally placed in front of the detector, eliminating all light except the wavelength of the selected gas molecules. The NDIR sensor described in this paper utilises a mid-infrared LED/PD combination, providing the wavelength selection necessary to prevent cross sensitivity without need for an optical filter. Sensor constituent parts and assembly are described in the sections that follow.

#### 2.1.1. LED/PD Combination

Photonics technology has been used [20,21,23] for producing LED sources and PD detectors operating at mid-infrared wavelengths of interest in gas detection. The technology is based on a pentanary AlGaInAsSb narrow bandgap III-V material combination. In the case of pentanary alloys, many compositions with identical band gaps and lattice parameters exist [23], providing scope to change alloy bandgap by changing composition, thereby providing a means to tune output wavelength [23]. Moreover, the existence of the additional degree of freedom for the control of material electro-physical properties by changing its chemical composition becomes a significant advantage. This offers a new approach [23,24] to control optical confinement and carrier leakage as compared to previously reported work [17–21], offering advantages in lifetime and stability compared to previous LED/PD structures [19,22].

**Figure 2.**
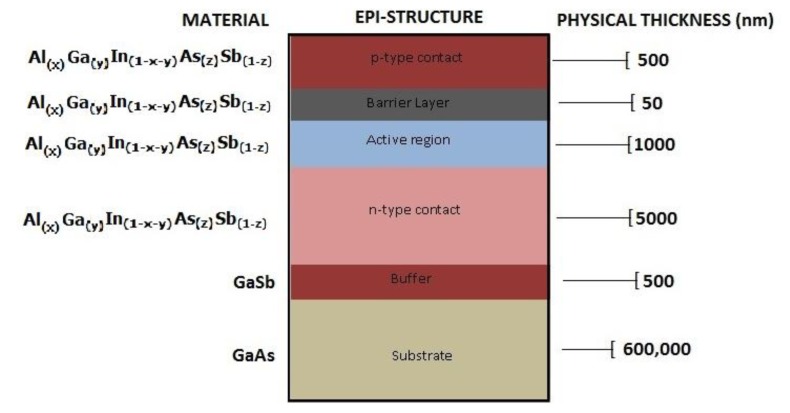
Schematic of narrow bandgap III-V AlGaInAsSb epi-grown structure.

Schematic of the generic epi-structure is shown in Figure 2. Epi-multilayer structure comprises a buffer layer to transition from substrate to epitaxial structure and an active region (light emission layer) with p and n contacts: p-type layers were doped with Be (1 × 10^18^ cm^−3^) and n-type layers were doped with Te (4 × 10^17^ cm^−3^). A barrier layer is introduced between active region and top p-type contact to further confine carriers. Photodiode structure is similar with an added absorption layer to increase detectivity.

Narrow bandgap III-V epitaxial growth of the LED and PD structures are produced using epitaxial growth under ultra-high vacuum from separate material sources, independently controlled to achieve the required material stochiometry. Shuttering of the sources and wafers being coated enable accurate layer thickness control of the LED and PD multilayer. Epitaxial growth process is fully automated and carried out in production scale equipment, providing required reproducibility and 4″ wafer throughput.

Previous work describing the usage of LEDs for gas sensors [19,22], evaluated early stage LED devices from research sources with shortfalls in device reliability. Gas Sensing Solutions (GSS) Ltd (Glasgow, Scotland, UK) has implemented robust high volume throughput manufacturing process & device design with standard commercial semi-conductor processing techniques. Specifically, Gas Sensing Solutions Ltd device processing addresses passivation within device manufacture (critical to long term stable performance and lifetime). GSS devices also utilise low temperature (<100 °C) sputter deposited high-permittivity (k) nitrides and/or oxides and the resulting mid-infrared LED/PD devices require no burn-in prior to use in sensors.

Accelerated lifetime testing has been carried out [25,26], providing device lifetimes >15 years. The accelerated life testing is based on testing of twenty four sensors powered continuously at 60 °C. LED/PD automated production processing is based on 4″ wafers, yielding *circa* 2,500 devices per wafer, providing LED/PD device costs compatible with use in gas sensors.

**Figure 3.**
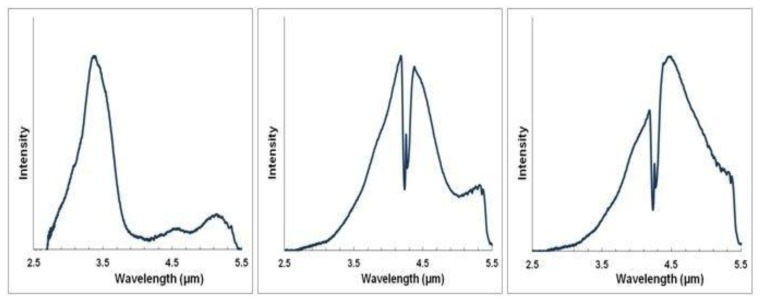
Tuned LED inputs to (**a**) 3.3 μm (methane); (**b**) 4.3 μm (CO_2_) and (**c**) 4.6 μm (CO).

**Figure 4.**
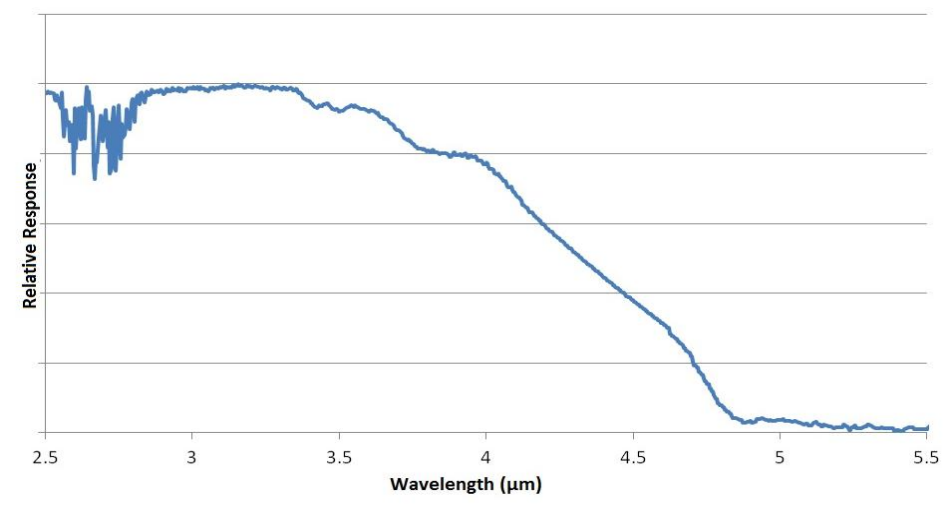
PD spectral response (feature at 2.8 μm is an artifact of water vapour in the measurement system).

Spectral characteristics of both LED and PD can be tuned by altering the ratio of the III-V material stoichiometry, enabling LED/PD spectral output to be matched to the specific gas absorption spectra shown in Figure 1. As such the LED and PD devices are designed with specific wavelengths as shown in Figures 3 and 4 respectively.

Figure 3(a–c) show measured LED tuned outputs to wavelengths overlapping gas absorption features in the mid infrared—3.3 (methane), 4.3 (CO_2_) and 4.5 μm (carbon monoxide), respectively [the absorption features exhibited in Figure 3(b,c) are due to CO_2_ gas within the measurement system]. Figure 4 shows PD spectral response.

**Figure 5.**
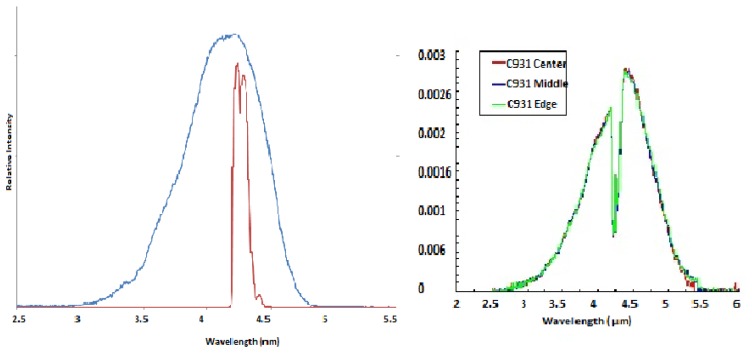
(**a**) Spectral profile for LED/PD combination (blue) and CO_2_ gas spectral absorption (red curve); (**b**) measured LED spectral output from centre/mid/edge wafer positions.

This technology produces LED and PD's tuned to a relatively narrow bandwidth. Used as a pair the bandwidth of the combined system is narrower still, as shown in Figure 5(a). This narrow bandwidth allows the LED and PD to be used without additional optical filtering, further reducing cost and simplifying the optical design. Figure 5(b) shows measured LED spectral emission characteristic from centre, mid and edge positions on a processed wafer, illustrating the high degree of control in the fabrication process across the wafer area [note the dip in measured emission in Figure 5(b) due to ambient CO_2_ in the measurement system].

**Figure 6.**
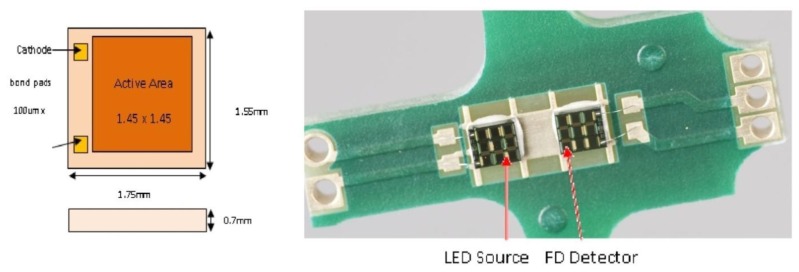
(**a**) LED and PD detector structure and (**b**) LED and PD detector mounted on Bridgeboard.

Figure 6(a) shows LED and PD structures and (b) LED/PD mounted on a bridgeboard.

LED and PD detector operating voltages are reduced to 2.5 to 3.3 V by enhanced impedance matching of the LEDs and PDs to the drive circuitry through segmentation of the LED and PD active areas [27,28]. Operating voltage at 2.5 V is well suited to interface with 5 V and 3.3 V electronics and powering from batteries and/or energy harvesting such as photovoltaics.

Previously reported results for LED usage within NDIR CO_2_ sensor [19], employed drive/signal processing electronics suited to thermal sources, consequently LED impedence matching to the electronics was not optimised. This paper focuses on LED/PD's tuned for sensing CO_2_ gas. Typical measured data for the mid infrared LED is provided in Table 1.

Typical PD detectivity (D*) is 2.0.10^8^ cm√Hz/W at room temperature. Work is underway within Gas Sensing Solutions Ltd to develop a methane sensor based on a LED/PD light source/detector.

#### 2.1.2. Optics and Signal Processing Optics

Detailed optical modelling of sensor sensitivity and noise has been carried out to identify necessary optical pathlength for CO_2_ gas sensor, covering the required concentration ranges (ambient to 100%), accuracies and data processing.

For a strictly monochromatic parallel light beam passing through an absorbing gas the absorption follows the Beer-Lambert equation:

$${\text{I}}_{\text{T}}={\text{Ioe}}^{-\text{lac}}$$

where I_T_ = *Transmitted intensity*, Io =*Incident intensity*; l = *path length*, a = *absorption coefficient*, c = *gas concentration*. Parameters I_T_, I_O_, and a are wavelength dependent. I_T_, I_O_ are temperature dependent and a pressure dependent.

Measured transmission (=I_T_/Io) is convolution of incident intensity [Figure 5(a)—LED spectral output × PD spectral output] and CO_2_ gas spectral absorption (provided from HITRAN database [29]). Transmission is shown in Figure 6 for two pathlengths (20 and 70 mm) as a function of CO_2_ gas concentration.

**Figure 7.**
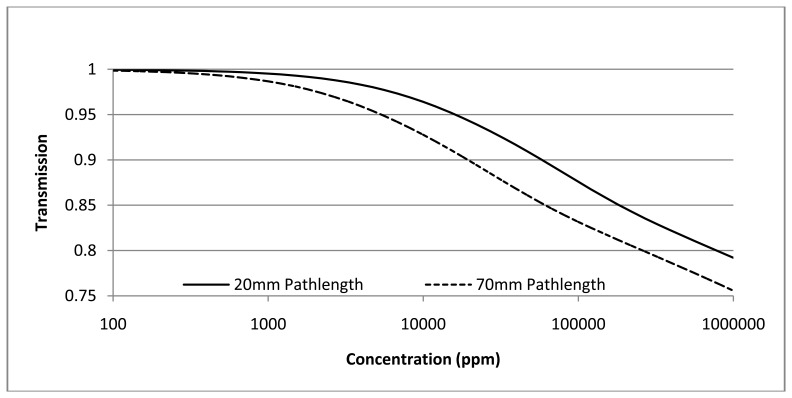
Modelled transmission as a function of gas concentration for two pathlengths.

Modeled data shown in Figure 7 includes convoluted spectral output of the measured LED/PD and CO_2_ gas spectral absorption [29]. Modelling shown in Figure 7 is for room temperature. Measured performance with temperature is described in Section 2.1.3. LED/PD spectral position is selected to ensure spectral temperature shift is accommodated over the sensor operating temperature range (extended operating temperature range is −25 °C to +50 °C).

**Figure 8.**
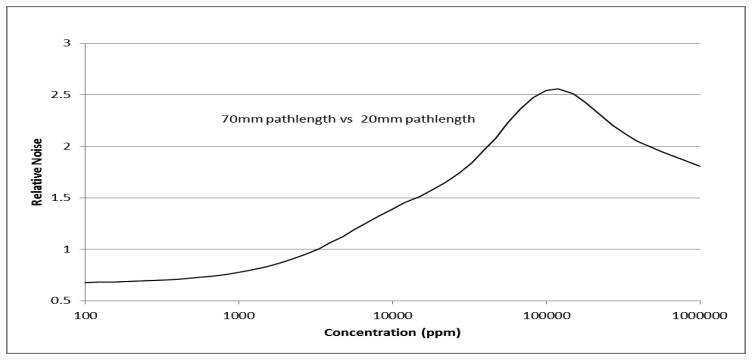
Modelled relative noise for two pathlengths [70 mm and 20 mm] as a function of CO_2_ gas concentration.

Modelled relative noise (Noise [pathlength 70 mm]/Noise [pathlength 20 mm]) is shown in Figure 8. Figures 7 and 8 show two pathlengths, 20 and 70 mm, are required to provide accurate measurement over ambient to 100% CO_2_ gas concentration. 20 mm pathlength is suited to concentration levels from 0.5 to 100% whilst 70 mm pathlength provides complementary required accuracy from 400 ppm to 0.5%.

Typical applications and required CO_2_ gas concentration measurement range provided in Table 2.

**Figure 9.**
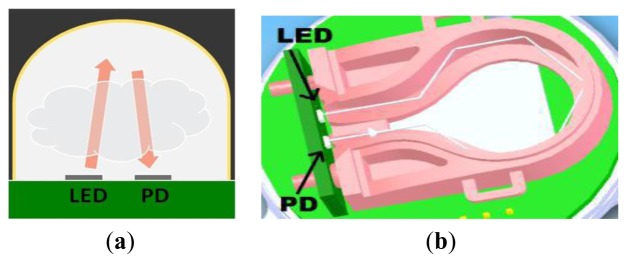
(**a**) 20 mm and (**b**) 70 mm optical configurations.

Figure 9 shows schematics of an optical configuration providing (**a**) 20 mm and (**b**) 70 mm pathlengths.

**Figure 10.**
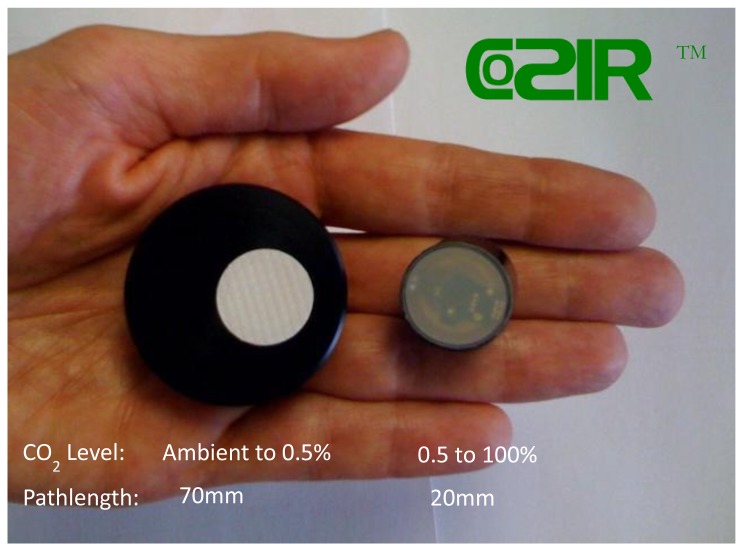
Production version of 70 (ambient) and 20 mm (wide range) pathlength LED/PD sensors—trademarked as COZIR™.

As Shown in Figure 9(a) 20 mm pathlength is a dome geometry with a single reflection, whilst 70 mm pathlength (Figure 9(b)) is achieved with a folded optic and multiple reflections between LED and PD. Both optical configurations are produced using injection moulded optics, reflective surfaces subsequently coated with a gold layer or protected aluminum to maximise mid-infrared optical throughput. No need for a separate optical filter greatly simplifies the optical design, with no subsequent requirement for collimated light. Figure 10 shows production version ambient and wide range sensors (trademarked as COZIR™).

Assembly requires no active alignment of LED/PD bridgeboard and uses low cost plastic optics.

##### Electronics & Signal Processing

Previous work describing use of LED's [19] for gas sensors, utilised signal processing designed for use with thermal sources and not optimised for use with significantly reduced LED power output. Work described in this paper utilises low cost compact signal processing techniques/available hardware, enabling successful measurements to be made using lower power output from LED and PD. Described signal processing provides sufficient processing speed and capacity for real time compensation of LED/PD wavelength and intensity change with temperature.

**Figure 11.**
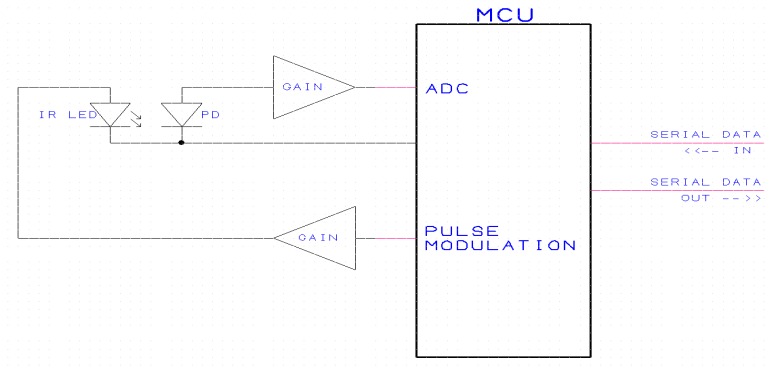
Signal processing configuration.

Figure 11 shows the signal processing scheme developed by Gas Sensing Solutions Ltd specifically designed for use with LED/PD devices. The Micro Controller Unit (MCU) periodically sends a very fast pulse train to the LED via a GAIN stage. The modulated mid-infrared light pulses fall on the PD detector.

Mid-infrared light level is detected by the PD and is amplified by a GAIN stage before being captured by the MCU's Analogue to Digital Convertor (ADC) thus making a digital version available for processing. The digital version of the IR light level is processed by the MCU which then provides a serial data output indicating the CO_2_ concentration present between the mid-infrared LED and the PD.

**Figure 12.**
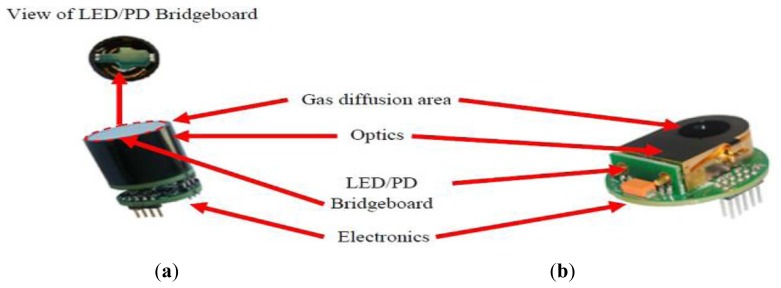
Assembled (**a**) wide range (actual size 20 mm diameter, 25 mm length including electronics) and (**b**) ambient sensors (note ambient is shown without protective cover. Size with cover 42 mm diameter, 15 mm thickness including electronics).

Figure 12 shows the assembly of production version (a) wide range (pathlength 20 mm) and (b) ambient (pathlength 70 mm) sensors.

#### 2.1.3. Calibration, Temperature, Noise & Cross Sensitivity Performance

##### Calibration and Temperature Compensation

LED/PD spectral characteristic is dependent on temperature. Temperature effects are compensated for through factory based calibration and real time compensation using signal processing. The sensor requires a two-point calibration, both carried out at the factory prior to dispatch. First, the sensor must be zeroed. This is performed by flowing a gas without carbon dioxide through the analyser and adjusting the zero on the analyser. Second, the span needs to be adjusted. A span gas containing an amount of carbon dioxide close to the concentration of carbon dioxide found in the sample gas should be used to span the analyser. The span gas is a known CO_2_ gas concentration.

As the LED/PD spectral characteristic changes with temperature, calibration also includes a procedure stepping through required temperature range and repeating zero/span CO_2_ gas concentration with temperature variation. Logged two-point gas concentration and temperature variation data is subsequently downloaded into the signal processing scheme shown in Figure 11.

**Figure 13.**
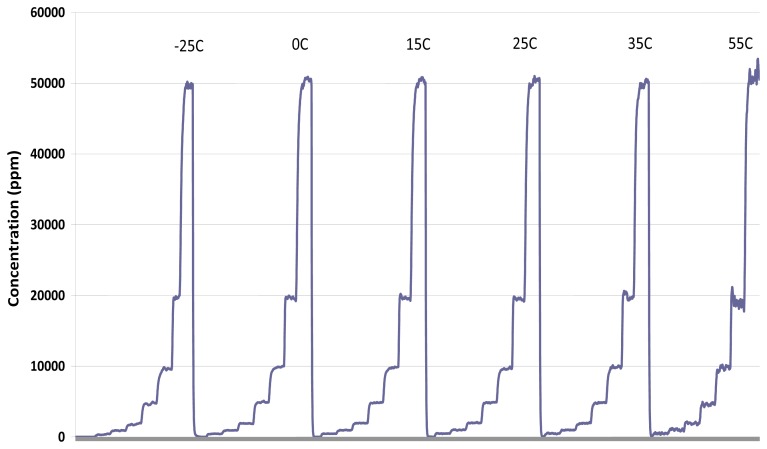
Temperature stability at 500 ppm, 1,000 ppm, 2,000 ppm, 5,000 ppm, 1%, 2%, 5% over temperatures −25, 0, 15, 25, 35, 55 °C.

Figure 13 shows temperature invariant performance over six temperatures (−25, 0, 15, 25, 35, 55 °C) with sensor stepped through seven CO2 concentration levels (500 ppm, 1,000 ppm, 2,000 ppm, 5,000 ppm, 1%, 2%, 5%) for an ambient sensor:

Calibration process within Gas Sensing Solutions Ltd is fully productionised and automated, capable of processing high volume sensor throughput.

**Figure 14.**
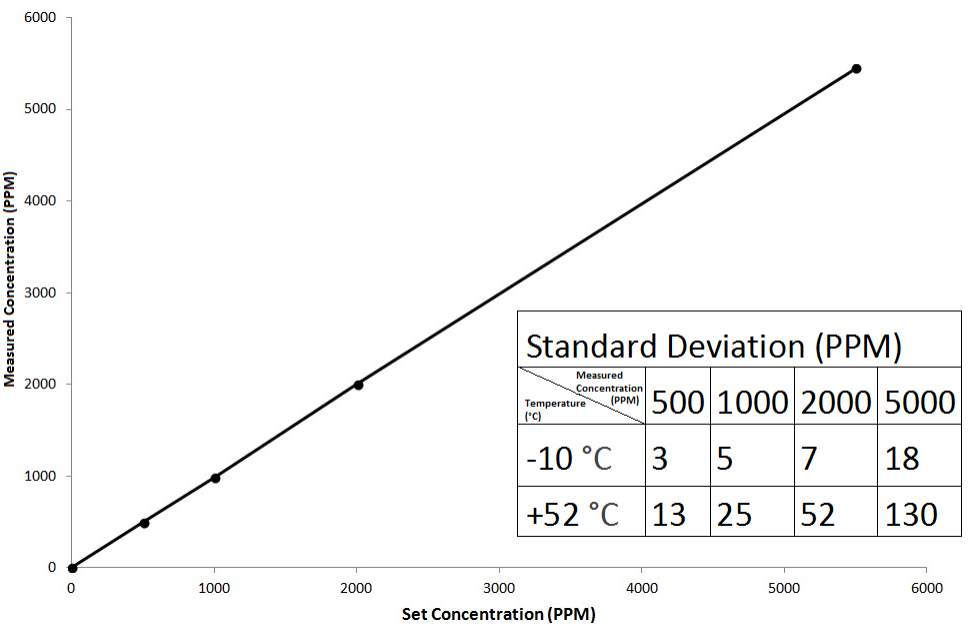
Linearised output (measured against set CO_2_ gas concentration) over temperatures −10, 8, 23, 33 and 52 °C for ambient sensor configuration. Measured standard deviation indicated.

Linearised signal output shown in Figure 14 with CO_2_ concentration is provided as either analogue or digital (UART R_X_…T_X_) output. Measured standard deviation at each set gas concentration for extremes in temperature (−10 to 52 °C) are shown in Figure 14 for linearized signal output. Figure 14 shows measured sensor gas concentration output (ambient) at five temperatures (−10, 8, 23, 33 and 52 °C) as a function of set CO_2_ concentration level delivered from a known gas concentration.

##### Noise Performance

**Figure 15.**
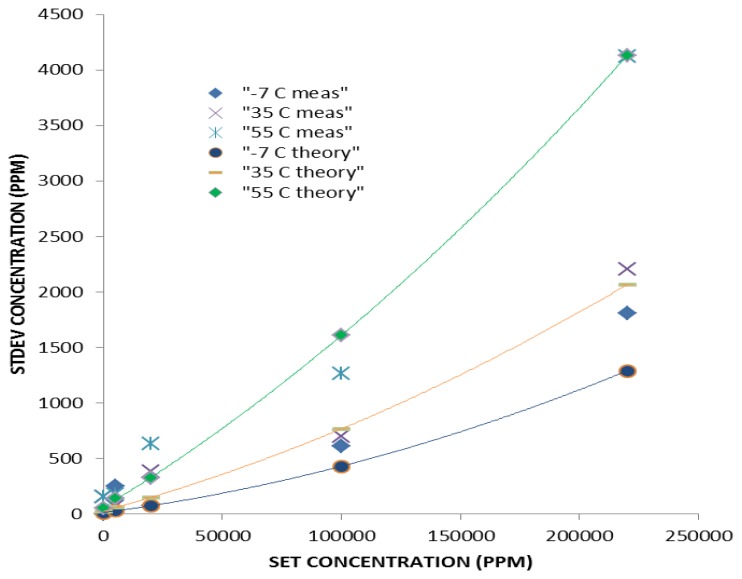
Measured standard deviation for three temperatures (−7, 25 and 55 °C) as a function of set concentration for wide range (pathlength 20 mm) sensor configuration. Modelled noise included.

Figure 15 shows measured standard deviation with set CO_2_ gas concentration for temperatures −7, 25 and 55 °C: wide range (pathlength 20 mm) configuration. Photodiode based detector comprises Johnson, dark current, quantisation and shot noise [30]. Modelled noise is shown in Figure 15.

Measured noise is also shown in Figure 15. Dominant noise contribution for temperature <20 °C is Johnson noise and temperature >20 °C dark current noise. Figure 15 shows noise increases with temperature, dominated by dark current noise with increasing temperature.

**Figure 16.**
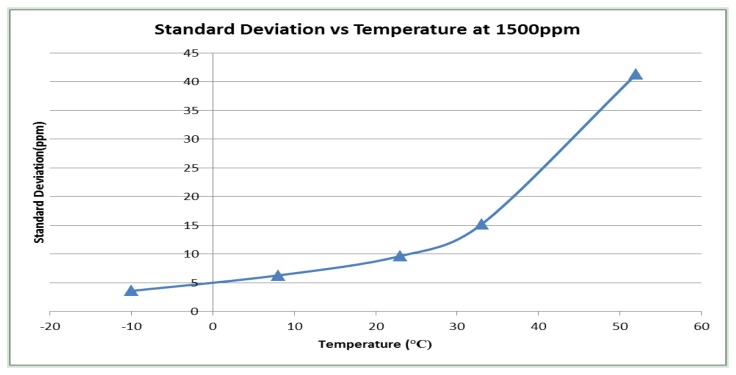
Measured standard deviation (of measured gas concentration output @ set gas concentration of 1,500 ppm) for ambient (pathlength 70 mm) with temperature (−10 to 55 °C).

Figure 16 provides measured standard deviation (of output gas concentration) for the ambient (pathlength 70 mm) configuration at 1,500 ppm for temperatures −10 to +55 °C.

Influence of noise with temperature can be reduced by increased measured signal averaging with time. Data shown in Figures 15 and 16 correspond to standard signal output from wide range and ambient configurations, based on averaged two measurements per second. Noise contributions and variation with temperature for ambient and wide range sensor configurations are similar as same LED/PD combination is used for in both types.

Table 3 shows ambient (pathlength 70 mm) variation in measured standard deviation (of measured gas concentration level) for three set concentration levels: standard operating condition for COZIR sensors, ie continuous power operation (result indicated for two averaged measurements taken over one second).

Pyroelectric detectors as used in standard thermal source/pyroelectric based NDIR sensors have three types of noise: thermal, dielectric and amplifier noise [30] with significant temperature dependency [31,32]. Overall noise and temperature dependency is comparable for the LED/PD configuration described in this paper and pyroelectric detectors [30].

##### Cross Sensitivity

**Figure 17.**
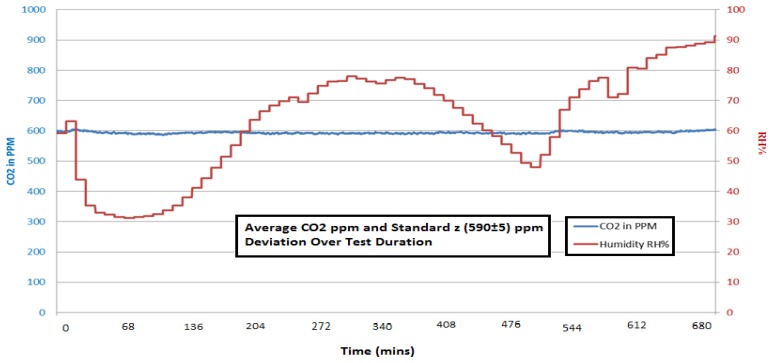
COZIR™ Ambient sensitivity assessment to water vapour for relative humidity (RH) variation from 30 to 90% at 25 °C.

Discrimination of the COZIR™ sensor has been evaluated for cross sensitivity to water vapour (absorption feature at 2.8 μm), methane (CH_4_−3.3 μm) and carbon monoxide (CO−4.6 μm). Figure 17 shows real time monitoring of a set CO_2_ level (590 ppm) at 25 °C as the humidity level is cycled from 30 to 90% over an 11.5 hour period—each change in humidity is allowed to settle over a ten minute period before humidity is subsequently step changed. Temperature/humidity combinations were chosen to ensure non-condensing conditions.

Cross sensitivity of COZIR™ ambient to CH_4_ and CO measured at 10,000 and 2,500 ppm respectively (values well in excess of trace amounts expected in air [33]), at 25 °C, show no variation with expected standard deviation. Nitrous oxide (4.5 μm–N_2_O) has not been tested, however minimal cross sensitivity is expected on the basis of peak absorption upshifted from CO_2_ [29], reduced absorption cross section compared with CO_2_, reduced LED/PD response at N_2_O absorption feature. Moreover, like CH4 and CO, trace amounts of N_2_O are expected in air [33].

## 3. Results and Discussion

### 3.1. LED/PD CO_2_ Sensor Performance

Performance of the two pathlength configurations is provided in Table 4.

Detailed specifications for COZIR™ ambient and wide range sensors are provided in references [34] and [35], respectively.

**Figure 18.**
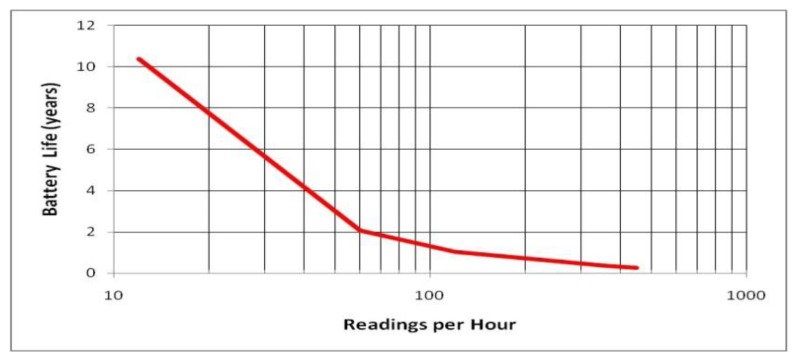
Battery life (AA sized battery) as a function of number of measurements per hour.

Performance shown in Figure 18 indicates > 10 year battery life at ≤ 10 measurements per hour, well within measurement frequency requirements for wireless deployment in building control applications and portable application in industrial safety and medical applications. Figure 18 accounts for energy required for one discrete measurement—the energy required to turn on the sensor from cold, warm up, take and report a measurement, and power down again.

### 3.2. Self-Powering Operation & Compatibility with Energy Harvesting

**Figure 19.**
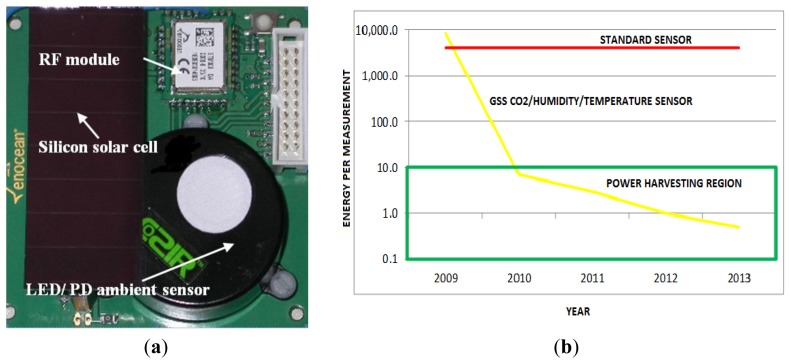
(**a**) Wireless self-powering LED/PD based CO_2_ COZIR™ ambient sensor; (**b**) Evolution of energy per measurement performance for the LED/PD CO_2_ and comparison with standard sensor and energy harvesting region.

Self-powered operation with wireless data transmission has also been demonstrated with a 67 × 28 mm silicon solar cell with 200 lux light level [36]—configuration shown in Figure 19(a). Self-powered operation enables the sensor to operate at one reading every 3 minutes.

To reduce and ultimately remove dependence on battery operation and increase time between battery changes (typically > 10 years), there is a developing need for cost effective energy harvesting solutions. Worldwide some 15 billion primary batteries are thrown away every year, all of which end up in landfill sites [37]. With the emergence of new low power sensor solutions the network communications landscape is rapidly changing from wired to wireless whereby all devices are to becoming connected, interoperable and require rapid deployment.

Evolution of energy per measurement (an important parameter in determining battery life/energy harvesting requirements) performance with development/production time for the COZIR™ LED/PD CO_2_ sensor is shown in Figure 19(b) [energy per measurement performance shown in Figure 19(b) incorporates humidity and temperature sensors, providing CO_2_/temperature/humidity in the one sensor platform—note that CO_2_ sensor energy per measurement dominates total energy per measurement shown in Figure 19(b)]. Less than 10 mJ per measurement is the upper limit for potential use of energy harvesting, based on performance assessment of initial prototypes based on silicon based photovoltaics [36], as shown in Figure 19(b).

### 3.3. Ultra-low Power Sensor Development with Schneider Electric Industries

**Figure 20.**
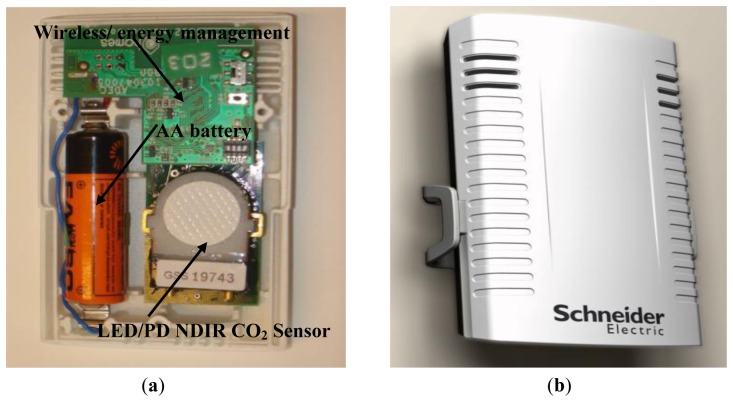
**ULP** LED/PD ambient sensor deployed in a Schneider Electric Industries single AA battery operated wireless CO2/ temperature/ humidity sensor platform for use in building control applications, (**a**) sensor platform showing integration of CO_2_ sensor (**b**) sensor in (a) with cover.

Figure 20(a,b) shows an ultra low power (ULP) wireless sensor module which contains a modified version of the sensor described in Section 2, a co-development with Schneider Electric Industries (SEI, Grenoble, France) [38]. The ULP CO_2_ sensor is combined with humidity and temperature sensors for use in indoor air quality monitoring and control within building applications.

This application uses the standard Gas Sensing Solutions (GSS) ambient sensor comprising LED, PD and optical chamber described in Section 2. However the drive electronics and signal processing have been optimized to minimize the energy required to power on for one measurement. The optimization focused on three main elements: reduction of start-up time, reduction of power wastage, and improvement in signal/noise (S/N) performance in the signal processing chain.

The start-up time was reduced to 5 ms by running startup processes in parallel, and by reducing the overall sensor capacitance. Reduction in the measurement cycle, the time when the LED is powered, had the dual effect of reducing the overall power requirements to the LED, and also reduced the time to first measurement.

Power wastage was minimized by intelligent switching of circuit elements so that resources such as amplifiers were powered only during the measurement cycle phase when they were required. The removal of capacitance from the sensor reduced energy lost between measurements, though placing greater demands on the external power management circuit to deliver the peak currents required by the sensor.

There is a trade-off between energy used and the S/N ratio—higher S/N can be used to provide a more stable reading, or to reduce the power consumption. The S/N ratio for this implementation was improved by increasing the operating frequency to reduce 1/f noise, by sharing the first stage amplification between two op amp, and by increasing the LED drive current, while reducing the pulse width to achive an overall reduction in energy per pulse.

Table 5 provides measured standard deviation (GSS/Schneider co-developed ultralow power ambient sensor configuration) for three set CO_2_ gas concentrations in cycled power mode for two energy per measurement values (1 and 3 mJ).

Expected improvement in standard deviation with increasing energy per measurement is observed at 900 and 1,500 ppm. No improvement is observed at 450 ppm [≈ambient], most probably due to variation in background actual ambient levels.

The SEI/GSS co-developed CO_2_/temperature/humidity ultra-low power sensor operates with two minutes between measurements, >ten year battery life (based on single AA battery), ±10 ppm resolution at 1,000 ppm, range ambient to 3,000 ppm, input voltage 3.6 V and compliant with solar cell powering [36].

### 3.4. LED/PD & Thermal Source/Pyroelectric CO_2_ Sensor Performance Comparison

Other NDIR sensors utilize thermal sources (tunsten lamps) and either pyroelectric or thermopile detectors. A disadvantage of thermal source-based NDIR sensors is that the sensor needs a significant time to warm-up to operating temperature following lamp switch-on due to the heat generated by the lamp warming up the entire sensor. There is a trade-off between lamp operating voltage, noise, lamp lifetime, warm-up time and operating power [39,40]. Optimum performance in relation to NDIR CO_2_ sensor [40] is to minimize lamp operating voltage, lower limit typically 2 V, with operating current of 38 mA and resulting average power consumption of 38 mW (50% duty cycle). Warm-up time is typically < 10 minutes to achieve temperature stability and hence signal equilibrium [40]—data based on an Alphasense Ltd IRC-A1 NDIR CO_2_ sensor relating to low voltage powered thermal source [40,41].

Table 6 provides a comparison of NDIR CO_2_ sensor performance (single channel devices with comparable accuracies as measured at standard temperature & temperature STP*) from a range of suppliers, mid infrared LED/PD (COZIR™: Gas Sensing Solutions Ltd; ULP: co-development Gas Sensing Solutions Ltd/ Schneider Electric Industries) and thermal source/pyro or thermopile detectors (Senseair, Sensecube, OptoSense, ELT, Teleaire). Note information provided in Table 6 is based on available public domain sensor performance data.

As explained in the introduction, applications in IAQ, DCV and portable CO_2_ measurement require low power consumption for continuous usage and/or minimal energy per measurement for cycled power/intermittent usage with consequent extended battery life and compatibility with energy harvesting strategies.

For continuous sensor operation difference in NDIR CO_2_ sensor power consumption between LED/PD and thermal source/pyroelectric is typically a factor of between ten to one hundred depending on specific NDIR (thermal source) sensor comparison.

Power cycled/intermittent usage requires sensor to be turn on from cold, warm up, take and report a measurement, and power down again in as short an interval as possible. For thermal source this is typically between one to ten minutes to achieve warm-up/stabilization [39], implying energy per measurement of typically 2,000 to 9,000 mJ (based on 40 mW to 150 mW power consumption and 1 minute stabilization time).

The COZIR™ mid-infrared LED/PD light source time power-up/measurement time is typically one second—dominated by electronic initialization, with operating voltage of 3.3V, average current 1.1 mA and power consumption 3.5 mW. Ultra-low power consumption LED/PD NDIR CO_2_ sensor co-developed with Schneider Electric Industries (described in Section 3.3) time is 50 ms. This implies energy per measurement of 1.0 mJ.

The improvement factor between LED/PD and thermal source NDIR CO_2_ sensor energy per measurement performance is typically 600 to 2,000.

As detailed in Section 3.2, this combination provides an energy per measurement enabling extended powering from batteries and compatibility with energy harvesting methods such as photovoltaics.

Whilst reported longevity is similar for thermal sources and LED's (typically > 15 years based on accelerated lifetime testing [25,26]), LED/PD combination is not prone to microphony and helium ingress, both of which can shorten incandescent bulb lifetime through filament degradation [41].

Standard NDIR CO_2_ sensors also require an optical filter to provide wavelength discrimination, increasing material cost, assembly complexity and requires collimated light for optimum optical filter operation. Tuneable LED/PD intrinsic wavelength selectivity for CO_2_ gas sensing dispenses with the need for optical filters. Moreover, LED/PD higher frequency pulsing performance and pulse shaping provides a basis for more effective signal processing schemes to maximise signal to noise ratio [42,43].

## 4. Conclusions/Outlook

A novel NDIR CO_2_ sensor based on use of mid-infrared light emitting diode/photodiode light source/detector devices is demonstrated. NDIR CO_2_ sensors incorporating the LED/PD light source detector devices provide less than 3.3 mW power consumption in continuous operation and for the ULP configuration ≈ 1 mJ measurement in pulsed mode—the energy required to turn on the sensor from cold, warm up, take and report a measurement, and power down again. Cycled powering performance is compatible with extended battery life and/or energy harvesting.

Work is underway to further improve the efficiencies of all components of the optical bench. A further reduction in power consumption by a factor of two is anticipated through improvements in LED and PD device design and optimization of the epitaxy. Further work is focused on improving optical coupling and on signal to noise improvements in the detection circuit LED/PD efficiencies, signal processing and optical throughput, thereby offering potential for further reduction in energy per measurement. Further reductions in energy per measurement will lead to increased usage of energy harvesting regimes such as dye sensitized [44] and organic photovoltaics [45], resulting in reduced dependency on battery operation.

Energy consumption levels in pulsed mode enable self-powered operation and wireless data communication using a silicon based solar cell [36]. Future work will investigate other photovoltaic power generation methods as silicon is not a viable energy harvesting route for building control as efficiencies are dramatically reduced under indoor lighting [typically 200 lux] conditions. Other energy harvesting strategies will include dye sensitized solar cells [44] and organic photovoltaics [45], offering higher efficiencies in lower diffuse lighting conditions and lower cost with potential for photovoltaic performance on flexible substrates.

The LED/PD approach also offers scope to enhance real time measurement of CO_2_ gas temporal characteristic through increased frequency cycled power operation. A version of the LED/PD NDIR CO_2_ sensor described in Section 2 is now in production (tradename SPRINTIR™) which offers twenty measurements per second [46]. Further developments of the SPRINTIR™ CO_2_ sensor are underway to align performance with emerging requirements in medical applications such as capnography and metabolic rate assessment [47].

The LED/PD approach can be extended to provide sensing of other gases such as methane, carbon monoxide and nitrous oxide. Development work is currently underway to extend the LED/PD approach to an NDIR methane gas sensor.

## Figures and Tables

**Table 1.** LED parameters and typical measurements.

**Parameter**	**Typical Measurement**
Peak Wavelength *λ*	4.3 μm+/−0.15 μm^1^
Bandwidth (50% points)	1 μm
Temperature Sensitivity of Peak wavelength	6.3 nm/°C
Drive voltage at 100 mA	2.0V +/−0.2V
Size	1.65 mm × 1.55 mm × 0.7 mm
Bond Pads	100 micron square Gold
Active Area	1.35 mm × 1.35 mm
Far Field Radiation Pattern	Lambertian
Optical Power	Approx 8 mW/cm^2^ at 25 °C, 50% duty cycle

**Table 2.** Applications and typical CO_2_ gas concentration levels.

**Application**	**Typical CO2 Gas Concentration Range**
Indoor Air Quality	Ambient to 5,000 ppm
Safety	Ambient to 5%
Horticulture	Ambient to 5,000 ppm
Combustion	Ambient–18%
Process control	Ambient–100%
Automotive	Ambient to 5,000 ppm
Medical	Ambient–5%

**Table 3.** Measured standard deviation for ambient sensor configuration for three set CO_2_ concentration levels (@ 28 °C). Continuous power mode with averaged two measurements over one second.

**Set Concentration (ppm)**	**Continuous Power (3.5 mW)**

	**St Dev (ppm (±%))**
450	6 (±0.7%)
900	9 (±0.5%)
1500	12 (±0.4%)

**Table 4.** Performance parameters (at STP) for COZIR™ wide range (20 mm) and ambient (70 mm).

**Parameter**	**Wide Range 20 mm Pathlength**	**Ambient 70 mm Pathlength**
Power (continuous)	≈ 3.5 mW	
Energy per measurement (pulsed)	12 mJ	
Power up stabilisation time	≈ 1 s	
Peak current	33 mA	
Average current	1.1 mA	
Input voltage	3.3 V	
Accuracy	±3 % (of reading)	
Standard CO_2_ gas concentration ranges	0 to 5, 20, 65 and 100%	0 to 2,000 ppm, 1 and 2%
T_90_	4 secs	30 secs
Operating temperature range (°C)	Standard: 0 to 50 °C; extended −25 °C to +50 °C	
Storage temperature (°C)	−30 to +70 °C	
Relative humidity	0 to 95% RH non-condensing	
Dimensions	20 mm × 25 mm	43 mm × 15 mm
Weight (grams)	10	20

**Table 5.** Measured standard deviation for ultralow power ambient sensor configuration for three set CO_2_ concentrations at 1 and 3 mJ per measurement.

**Set Concentration (ppm)**	**Energy per Measurement (mJ)**	

	**1**	**3**

	**St Dev (ppm (±%))**	
450	18 (±2.0%)	18 (±2.0%)
900	40 (±2.2%)	25 (±1.4%)
1500	78 (±2.6%)	32 (±1.1%)

**Table 6.** Comparison of performance data for similar accuracy single channel NDIR CO_2_ sensors.

**Sensor**	**Manufacturer**	**Measurement Range**	Accuracy (STP)*	**Power Supply Voltage (V)**	**Power** ** **Cons mW**	**Current Cons mA (Avg)**	**Warm-up Time**
**LED/PD**							
COZIR Ambient	GSS	Ambient–5,000 ppm	±50 ppm +/−3% of reading	3.2 to 5 V	3.3	<1.5	1.2 s (1st Reading)
COZIR Wide Range	GSS	0.5–100%	±70 ppm +/−5% of reading	3.2 to 5 V	3.3	<1.5	1.2 s (1st Reading)
ULP***	GSS/SEI	Ambient to 3,000 ppm	±50 ppm +/−3% of reading	3.6 V	0.07	<0.025	5 ms
**Thermal Source**/**Pyro or Thermopile Detector**							
CO_2_ Engine K30 FR	SenseAir	Ambient–5,000 ppm	±70 ppm +/−5% of reading	4.5 to 14 V	315	70	60 s
KCD-AN 100x	SenseCube	Amb–10,000 ppm	±4% FS +3% of reading	8 to 14 V	560	70	Undisclosed
OS IAQ Sensor VS	OptoSense AS	Ambient–2,000 ppm	±<50 ppm	16 to 38 V	500	30	<60 s
S-100	TCC ELT	Ambient–10,000 ppm	±30 ppm +/−5% of reading	5 V	100	20	Undisclosed
S8	SenseAir	Ambient–10,000 ppm	±70 ppm +/−3% of reading	4.5 to 5.25 V	135	30	Undisclosed
Telaire 6613	GE Telaire	Ambient–2,000 ppm	±30 ppm +/−5% of reading	5 V	165	19	<120 s (1st Reading)

* Standard Temperature & Pressure (STP);

** care has to be taken when interpreting power consumption on the basis of used duty cycle.

*** Cycled/ intermittent operation. as per Section 3.3.
